# Prebiotic/probiotic supplementation resulted in reduced visceral fat and mRNA expression associated with adipose tissue inflammation, systemic inflammation, and chronic disease risk

**DOI:** 10.1186/s12263-022-00718-7

**Published:** 2022-11-28

**Authors:** Brian K. McFarlin, Elizabeth A. Tanner, David W. Hill, Jakob L. Vingren

**Affiliations:** 1 Applied Physiology Laboratory, University of North Texas, Denton, TX 76203 USA; 2 Department of Biological Sciences, University of North Texas, Denton, TX 76203 USA

**Keywords:** VAT, DXA, Body composition, Nanostring, Weight stable, Metabolically unhealthy

## Abstract

**Background:**

Prebiotic/probiotic supplementation represents a viable option for addressing elevated systemic inflammation and chronic disease risk in overweight individuals. The purpose of this study was to determine if 90 days of prebiotic/probiotic supplementation could alter mRNA responsible for inflammation and chronic disease risk in weight-stable overweight adults. Nanostring mRNA analysis (574 plex) was used to survey targets associated with adipose tissue inflammation, systemic inflammation, and chronic disease risk. All protocols were approved by the University IRB, and participants gave written informed consent. Participants were randomly assigned to either placebo (*N* = 7; rice flour) or combined (*N* = 8) prebiotic (PreticX® Xylooligosaccharide; 0.8 g/day; ADIP) and probiotic (MegaDuo® *Bacillus subtilis* HU58 and *Bacillus coagulans* SC-208; billion CFU/day) supplementation. Participants were diverse population of healthy individuals with the exception of excess body weight. Measurements were made at baseline, 30, 60, and 90 days. Whole-body DXA scans (GE iDXA®; body composition) and blood 574-plex mRNA analysis (Nanostring®) were used to generate primary outcomes. Significance was set to *p* < 0.05 and adjusted for multiple comparisons where necessary.

**Results:**

Compared to placebo, prebiotic/probiotic supplementation was associated with a 35% reduction in visceral adipose tissue (VAT; *p* = 0.002) but no change in body weight or overall percent body fat. Prebiotic/probiotic supplementation resulted in significant (*p* < 0.05), differential expression of 15 mRNA associated with adipose tissue inflammation (GATA3, TNFAIP6, ST2, CMKLR1, and CD9), systemic inflammation (LTF, SOCS1, and SERPING1), and/or chronic disease risk (ARG1, IL-18, CCL4, CEACAM6, ATM, CD80, and LAMP3). We also found 6 additional mRNA that had no obvious relationship to three previous biological functions (CSF1, SRC, ICAM4CD24, CD274, and CLEC6A).

**Conclusion:**

The key findings support that 90-day prebiotic/probiotic supplementation may be associated with reduced adipose tissue inflammation, reduced systemic inflammation, and reduced chronic disease risk. Combined with the unexpected finding of reduced VAT, this intervention may have resulted in improved overall health and reduced chronic disease risk.

## Background

Prebiotic/probiotic supplementation has been linked to improvement in a variety of health conditions and chronic disease states, including reduced severity of irritable bowel syndrome, reduced risk of type 2 diabetes mellitus, and reduced risk of cardiovascular disease [1]. Given that most chronic disease states have an inflammatory etiology, prebiotic/probiotic supplementation may represent a useful treatment alternative to westernized approaches. The primary mechanism of chronic disease reduction with prebiotic/probiotic is via immune system modulation and reduced peripheral/systemic inflammation [2]. While these supplements are frequently used as part of a comprehensive intervention, they are also known to exert independent effects [2, 3]. Previous research has also reported that it is possible to shift an individual from a metabolically unhealthy to a metabolically healthy phenotype without actually changing weight [4, 5]. Furthermore, the ability to uncouple body weight from metabolic health has implications not only for weight loss but also for individuals struggling to maintain weight loss [4, 5]. Typically, treatment of obesity involves a comprehensive lifestyle intervention with many components (i.e. calorie restriction, exercise intervention, behavioral treatment) [6].

Prebiotic/probiotic supplementation has known anti-inflammatory effects [7–11]; however, we speculate that reduced inflammation may have the potential to improve metabolic health, independent of weight loss. Our laboratory has previously reported that a multi-strain probiotic supplement reduced serum inflammatory cytokines and indices of dietary endotoxemia in weight stable adults [8]. Our previous study was limited to a 30-day intervention, so the present study would expand that timeline by examining changes over a 90-day period in free-living adults. The primary purpose of this study was to determine if 90 days of prebiotic/probiotic supplementation could alter mRNA responsible for inflammation and chronic disease risk in in weight stable overweight adults. A secondary purpose was to determine if there were any changes in the relative distribution of body fat.

## Results

### Body composition and anthropometrics

A summary of participant characteristics and baseline entry information is presented in Table 1. There was no significant percent change in total body mass, fat mass, or percent body fat in either group at baseline or during the 90 days (Table 2). This supports the notion that the participants were weight stable. The prebiotic/probiotic group had a significant (*p* = 0.002) reduction in visceral adipose tissue (VAT) at 60 days (37%) and 90 days (35%). In comparison, placebo had a slight, nonsignificant increase in VAT during course of the study.

**Table 1:** Participant characteristics

Variable	Placebo (*N* = 7)	Supplement (*N* = 8)
Sex		
(F)	5	3
(M)	2	5
Age		
(Y)	21 ± 3	22 ± 7
Height (cm)	166 ± 6	169 ± 13
BMI (kg/m^2^)	27.6 ± 2.8	30.8 ± 3.5
Resting metabolic rate (kcal/day)	1603 ± 100	1871 ± 110

Probiotic Supplement (MegaDuo® Bacillus subtilis HU58 and Bacillus coagulans SC-208; 3 × 109 CFU/day; Microbiome Labs, LLC; Phoenix, AZ, USA). Values represent the mean ± standard error of the mean (SEM). Variable were compared between combined supplementation with prebiotic (PreticX® Xylooligosaccharide; 0.8 g/d; ADIP; City of Industry, CA) and probiotic (MegaDuo® Bacillus subtilis HU58 and Bacillus coagulans SC-208; 3 Billion CFU/d; NuScience Trading LLC; Phoenix, AZ) and Placebo (rice four) in free living overweight individuals over 90-d. No significant differences detected for participant characteristics between condition groups

**Table 2:** Participant body composition

Condition	Variable	Baseline (0 day)	30 days	60 days	90 days
Placebo (*N* = 7)	Mass (kg)	82.3 ± 7.8	81.8 ± 7.7	82.0 ± 7.5	82.2 ± 7.3
	Fat mass (kg)	33.1 ± 3.9	32.6 ± 3.7	33.0 ± 4.0	33.2 ± 4.0
	VAT (kg)	1.1 ± 0.4	1.1 ± 0.4	1.2 ± 0.4	1.1 ± 0.4
	VAT (∆)		−0.02	0.07	0.02
	Body fat %	41.3 ± 1.8	41.0 ± 2.0	41.2 ± 2.1	41.4 ± 2.1
Supplement (*N* = 8)	Mass (kg)	93.1 ± 8.3	92.6 ± 7.3	92.8 ± 7.7	92.8 ± 7.7
	Fat mass (kg)	35.4 ± 4.0	34.6 ± 3.5	35.2 ± 3.8	35.4 ± 3.8
	VAT (kg)	1.2 ± 0.4	1.0 ± 0.2	0.8 ± 0.1	0.8 ± 0.2
	VAT (∆)		−0.08	−0.23*†	−0.26*
	Body fat %	39.3 ± 2.6	38.9 ± 2.7	39.1 ± 2.5	39.3 ± 2.7

Values represent the mean ± standard error of the mean (SEM). Body composition variables were compared between combined supplementation with prebiotics (PreticX® Xylooligosaccharide; 0.8 g/day; ADIP; City of Industry, CA, USA) and probiotic (MegaDuo® *Bacillus subtilis* HU58 and *Bacillus coagulans* SC-208; 3 billion CFU/day; NuScience Trading LLC; Phoenix, AZ, USA) and placebo (rice four) in free living overweight individuals at baseline (0 day), 30 days, 60 days, and 90 days of supplementation. †Represents significantly greater reduction in VAT than placebo at 60 days (*p* < 0.05). *Represents significant reduction from 30 days (*p* < 0.05)

### Overview of mRNA expression analysis

**Fig. 1:**
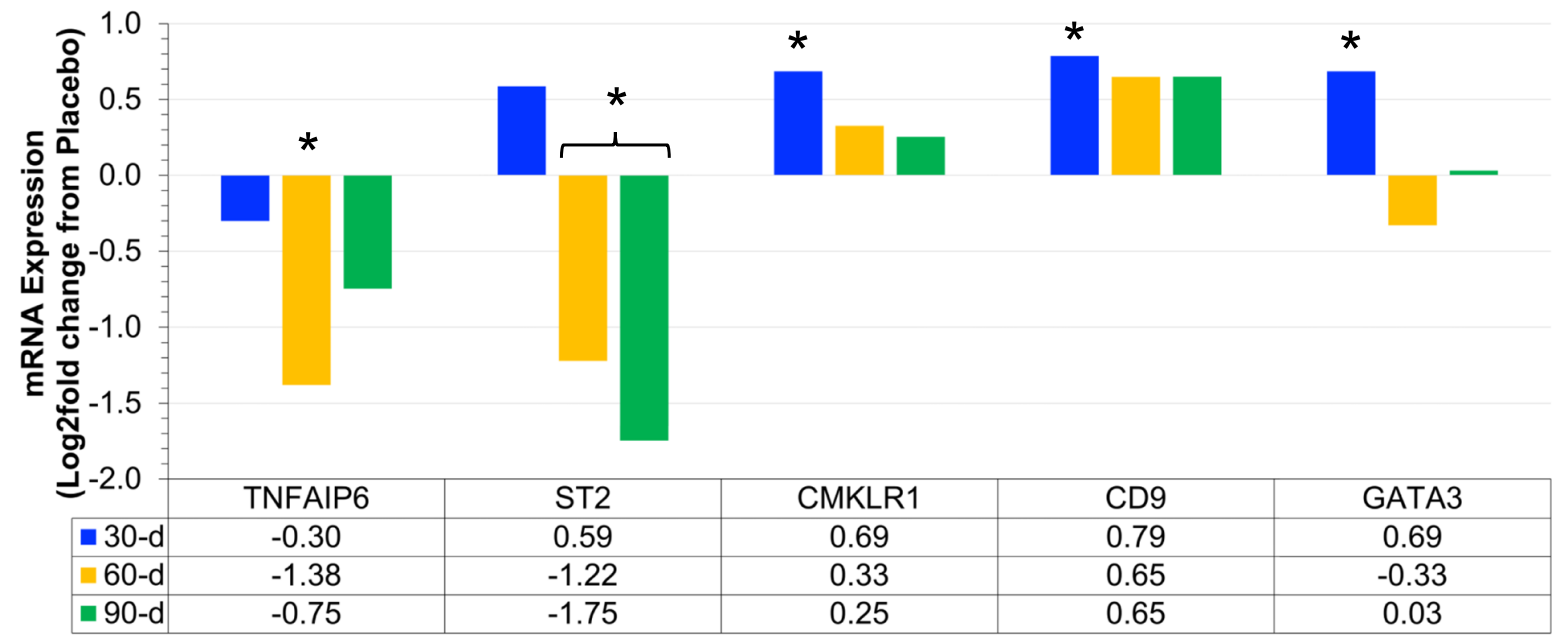
Adipose tissue inflammation associated mRNA (Log2 fold difference from placebo) was compared as a function of either combined (*N* = 8) supplementation with prebiotic (PreticX® Xylooligosaccharide; 0.8 g/day; AIDP; City of Industry, CA, USA) and probiotic (MegaDuo® *Bacillus subtilis* HU58 and *Bacillus coagulans* SC-208; 3 billion CFU/d; Microbiome Labs, LLC; Phoenix, AZ, USA) and placebo (*N* = 7; rice four). Free living overweight individuals reported to the baseline (0 day), 30 days, 60 days, and 90 days for the collection of PAXgene blood samples. Total RNA was isolated and analyzed using a 574-plex mRNA Immunology array (Nanostring® nCounter). ROSALIND® Advanced Analysis Software was used to calculate Log2 fold difference from placebo for each timepoint. Significance was set at *p* < 0.05 and adjusted for multiple comparisons to identify mRNA whose expression differed significantly as a function of prebiotic/probiotic (indicated as *)

**Fig. 2:**
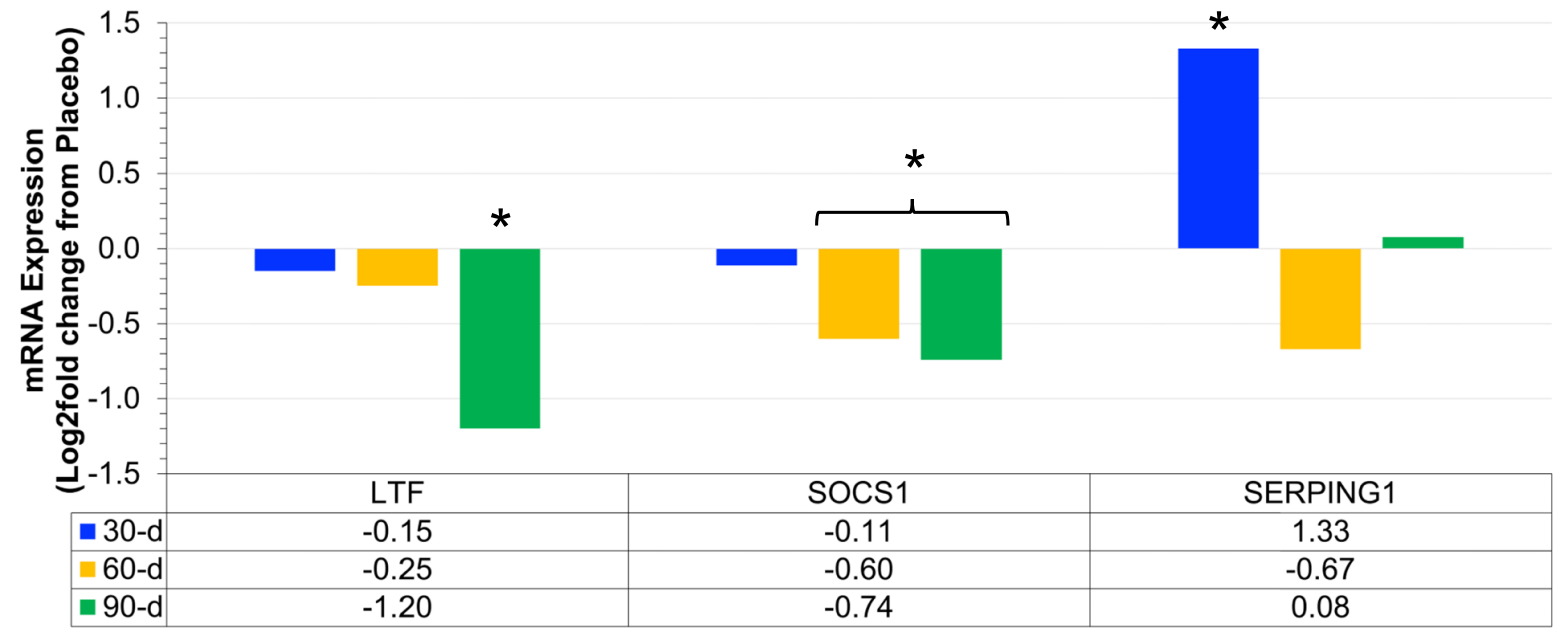
Differential expression of mRNA associated with systemic inflammation (Log2 fold difference from placebo) was compared as a function of either combined (*N* = 8) supplementation with prebiotic (PreticX® Xylooligosaccharide; 0.8 g/day; AIDP; City of Industry, CA, USA) and probiotic (MegaDuo® *Bacillus subtilis* HU58 and *Bacillus coagulans* SC-208; 3 billion CFU/d; Microbiome Labs, LLC; Phoenix, AZ, USA) and placebo (*N* = 7; rice four). Free living overweight individuals reported to the baseline (0 day), 30 days, 60 days, and 90 days for the collection of PAXgene blood samples. Total RNA was isolated and analyzed using a 574-plex mRNA Immunology array (Nanostring® nCounter). ROSALIND® Advanced Analysis Software was used to calculate Log2 fold difference from placebo for each timepoint. Significance was set at *p* < 0.05 and adjusted for multiple comparisons to identify mRNA whose expression differed significantly as a function of prebiotic/probiotic (indicated as *)

**Fig. 3:**
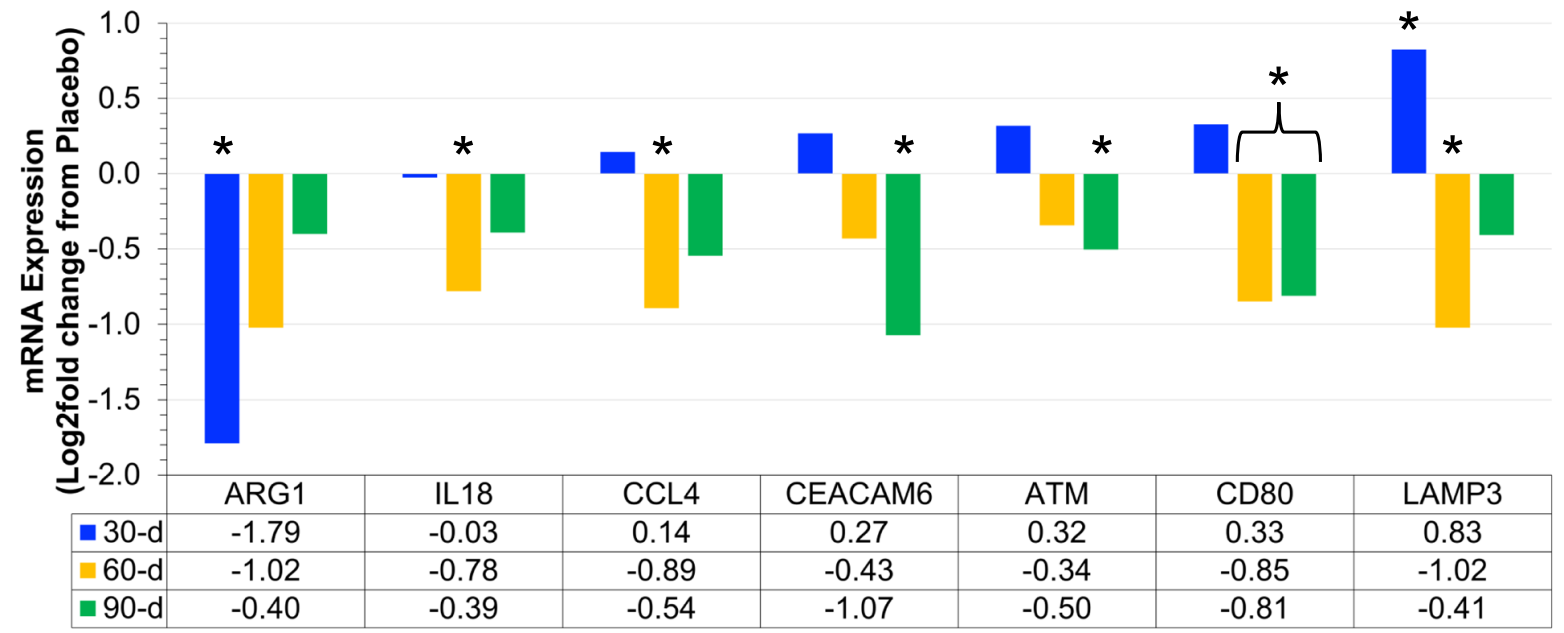
Differential expression of mRNA associated with chronic disease (Log2 fold difference from placebo) was compared as a function of either combined (*N* = 8) supplementation with prebiotic (PreticX® Xylooligosaccharide; 0.8 g/day; AIDP; City of Industry, CA, USA) and probiotic (MegaDuo® *Bacillus subtilis* HU58 and *Bacillus coagulans* SC-208; 3 billion CFU/d; Microbiome Labs, LLC; Phoenix, AZ, USA) and placebo (*N* = 7; rice four). Free living overweight individuals reported to the baseline (0 day), 30 days, 60 days, and 90 days for the collection of PAXgene blood samples. Total RNA was isolated and analyzed using a 574-plex mRNA Immunology array (Nanostring® nCounter). ROSALIND® Advanced Analysis Software was used to calculate Log2 fold change using the placebo samples for each timepoint. Significance was set at *p* < 0.05 and adjusted for multiple comparisons to identify mRNA whose expression differed significantly as a function of prebiotic/probiotic (indicated as *)

**Fig. 4:**
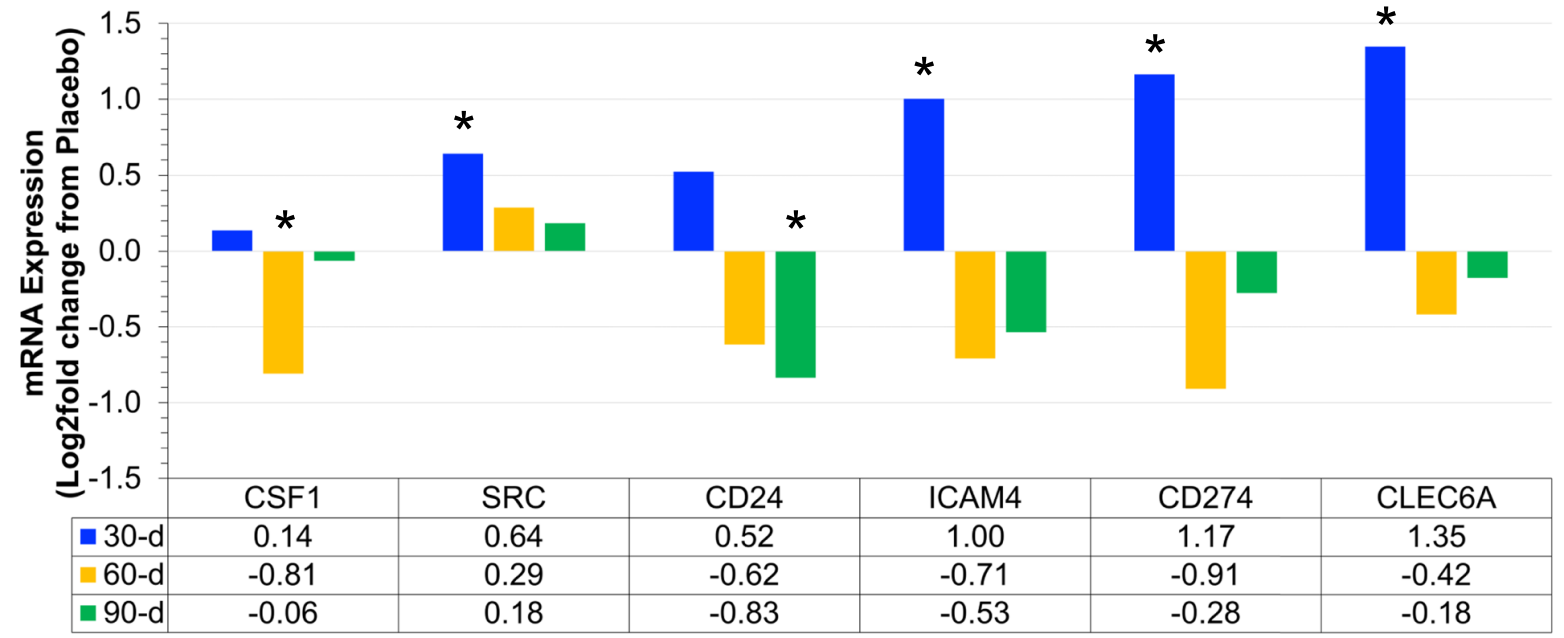
Differential expression of novel mRNA (Log2 fold difference from placebo) was compared as a function of either combined (*N* = 8) supplementation with prebiotic (PreticX® Xylooligosaccharide; 0.8 g/day; AIDP; City of Industry, CA, USA) and probiotic (MegaDuo® *Bacillus subtilis* HU58 and *Bacillus coagulans* SC-208; 3 billion CFU/d; Microbiome Labs, LLC; Phoenix, AZ, USA) and placebo (*N* = 7; rice four). Free living overweight individuals reported to the baseline (0 day), 30 days, 60 days, and 90 days for the collection of PAXgene blood samples. Total RNA was isolated and analyzed using a 574-plex mRNA Immunology array (Nanostring® nCounter). ROSALIND® Advanced Analysis Software was used to calculate Log2 fold change using the placebo samples for each timepoint. Significance was set at *p* < 0.05 and adjusted for multiple comparisons to identify mRNA whose expression differed significantly as a function of prebiotic/probiotic (indicated as *)

**Fig. 5:**
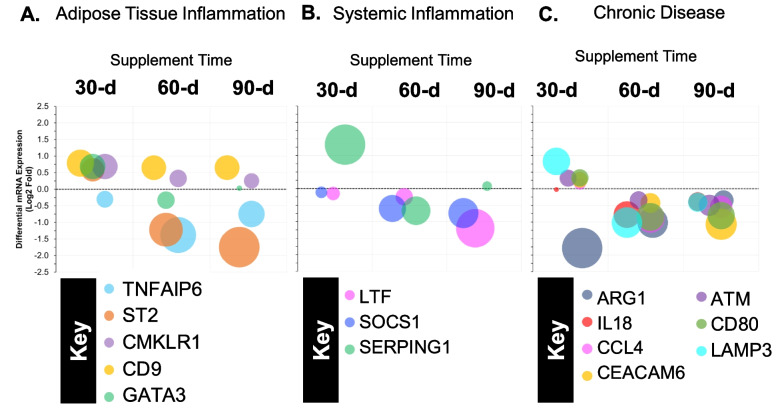
Differential expression of mRNA (Log2 fold difference from placebo) grouped according to biological pathway. **A** Adipose tissue inflammation. **B** Systemic inflammation, and **C** chronic disease. The prebiotic + probiotic supplement (PreticX® Xylooligosaccharide; 0.8 g/day; AIDP; City of Industry, CA, USA; MegaDuo® *Bacillus subtilis* HU58 and *Bacillus coagulans* SC-208; 3 billion CFU/day; Microbiome Labs, LLC; Phoenix, AZ, USA) was compared to a placebo (rice flour) group. A random, double-blind approach was used to assign participants to each condition. The dotted horizontal line represents no change relative to placebo at a given timepoint. Values above the dotted horizontal line are an increase in differential expression relative to placebo, and values below the dotted horizontal line represent a decrease in differential expression relative to placebo. The size of each spot is proportional to the Log2 change in differential mRNA expression from placebo

**Fig. 6:**
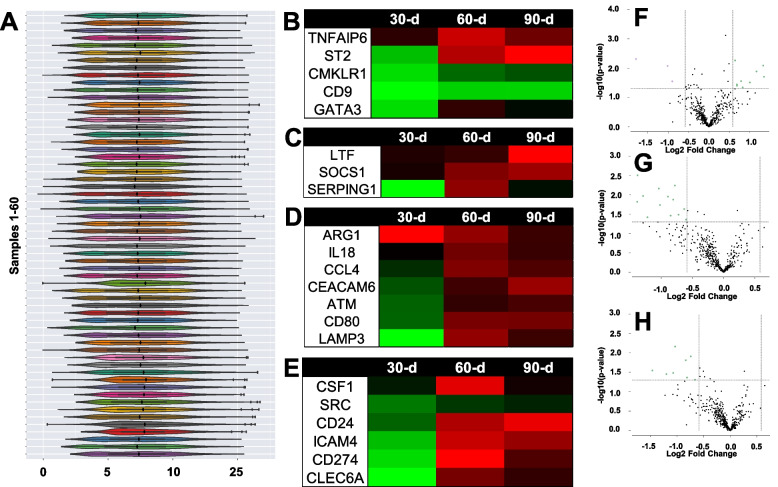
Human blood samples were analyzed for mRNA expression using a Nanostring nCounter array (Seattle, WA, USA). **A** demonstrates the relative gene expression for samples collected in this study using standard violin plots. **B** demonstrates an overall heat map response of all outcome variables in the study. An associated legend has been included to the right of Fig. 6B to demonstrate what the colors above the heat map represent. This included participant ID, Nanostring cartridge ID, condition, and time point. **C** demonstrates the relative expression of positive (*N* = 6) and negative (*N* = 8) controls across a range of samples analyzed in the study. This quality control analysis demonstrates that the expected values (positive controls) were linear and expressed at different levels across all samples. Positive controls were selected based on different levels of expression. In contrast, none of the samples was determined to have high levels of the negative controls. The conclusion is that analysis of the samples in the present study was within manufacture recommended parameters. **D**, **E**, and **F** demonstrate a volcano plot of genes with significant differential expression at 30, 60, and 90 days, respectively

Our initial analysis identified 21 mRNA that were differentially expressed in prebiotic/probiotic compared to placebo at 30 days (Fig. 6F), 60 days (Fig. 6G), and/or 90 days (Fig. 6H). After determination of significance, we conducted a systematic review of the existing literature and Nanostring pathway annotations to group significant mRNA. Of the 21 significant mRNA, we found 15 that could be directly associated with adipose tissue inflammation (Section 3.3; Figs. 1 and 6B; GATA3, TNFAIP6, ST2, CMKLR1, and CD9), systemic inflammation (Section 3.4; Fig. 2; Fig. 6C; LTF, SOCS1, and SERPING1), and/or chronic disease risk (Section 3.5; Figs. 3 and 6D; CCL4, IL-18, ARG1, ATM, CD80, CEACAM6, and LAMP3). The remaining 6 mRNA (Section 3.6; Figs. 4 and 6E; CSF1, SRC, ICAM4, CD24, CD274, and CLEC6A) may be novel responders affected by prebiotic/probiotic supplementation but not associated with any of the above biological pathways. To allow the reader to more easily compare the changes of 15 mRNA directly linked to inflammation and chronic disease, we created Fig. 5 to demonstrate both expression direction (i.e., positive or negative) and expression magnitude (i.e., log2 fold difference). Additional representation of global changes is represented in Fig. 6.

### Adipose tissue inflammation mRNA expression

Prebiotic/probiotic resulted in a significant, differential mRNA expression from placebo (Figs. 1 and 6B) for GATA3 (up at 30 days; *p* = 0.036), TNFAIP6 (down at 60 days; *p* = 0.003), ST2 (down at 60 days; *p* = 0.038), CMKLR1 (up at 30 days; *p* = 0.040), and CD9 (up at 30 days; *p* = 0.028). These 5 mRNA have been associated with adipose tissue inflammation, which is known to influence chronic, systemic inflammatory levels [12–19].

### Systemic inflammation mRNA expression

Prebiotic/probiotic resulted in a significant, differential mRNA expression from placebo (Figs. 2 and 6C) for LTF (down at 90 days; *p* = 0.035), SOCS1 (down at 60 days; *p* = 0.044; down at 90 days; *p* = 0.012), and SERPING1 (up at 30 days; *p* = 0.008). These 3 mRNA have been previously associated with the accumulation and/or management of systemic inflammation [1, 5, 6, 20, 21].

### Chronic disease mRNA expression

Prebiotic/probiotic resulted in a significant, differential mRNA expression from placebo (Figs. 3 and 6D) for CCL4 (down at 60 days; *p* = 0.011), IL-18 (down at 60 days; *p* = 0.006), ARG1 (down at 30 days; *p* = 0.005), ATM (down at 90 days; *p* = 0.030), CD80 (down at 60 days; *p* = 0.035; down at 90 days; *p* = 0.043), CEACAM6 (down at 90 days; *p* = 0.033), and LAMP3 (up at 30 days; *p* = 0.046; down at 60 days; *p* = 0.018). These 7 mRNA have been linked to the onset and progression of cardiovascular disease, type 2 diabetes mellitus, and other chronic inflammatory diseases [22–26].

### Novel/unknown mRNA expression

Prebiotic/probiotic resulted in a significant, differential mRNA expression from placebo (Figs. 4 and 6E) for CSF1 (down at 60 days; *p* = 0.013), SRC (up at 30 days; *p* = 0.005), ICAM4 (up at 30 days; *p* = 0.031), CD24 (down at 90 days; *p* = 0.015), CD274 (up at 30 days; *p* = 0.013), and CLEC6A (up at 30 days; *p* = 0.020). After reviewing the literature and Nanostring annotated pathways, we were unable to group these 6 mRNA with others because they have no clear role in adipose tissue inflammation, systemic inflammation, or chronic disease.

## Discussion

The key finding of the present study was that 90 days of prebiotic/probiotic supplementation resulted in 15 mRNA being differentially expressed from placebo. These mRNA were associated with adipose tissue inflammation, systemic inflammation, and/or chronic disease risk. Collectively, these changes may be classified as anti-inflammatory effects. We also had an unanticipated finding of an overall 36% reduction in VAT with prebiotic/probiotic supplementation compared to placebo. Previous studies have reported that similar reductions as in VAT converted obese individuals from a metabolically unhealthy to a metabolically healthy phenotype [4, 5]. Reduced VAT combined with the significant differential mRNA changes leads us to speculate that overall health may also have been improved with prebiotic/probiotic supplementation. To our knowledge, the present study is may be first to report a potential conversion to a metabolic healthy phenotype with prebiotic/probiotic supplementation.

Adipose tissue inflammation is a well-documented side effect of weight gain and obesity [13, 15–17, 19]. Prebiotic/probiotic supplementation had pronounced impacts on differential expression of three mRNA linked to adipose tissue (GATA3, TNFAIP6, and ST2) and affected two others to a lesser degree (CMLKR1 and CD9). GATA3 has both anti-adipogenic and anti-inflammatory effects [18], while TNFAIP6 has anti-inflammatory effects [17] on peripheral adipose tissue. Interleukin 1 receptor-like 1 (ST2) promotes adipose tissue inflammation when it is ligated by IL-33 [16, 19]; ST2 expression is elevated in the adipose tissue of severely obese humans compared to normal weight individuals. Regardless, controlling propensity for weight gain and reducing adipose tissue inflammation translate to reduced chronic disease risk [12, 16]. In the present study, we found that GATA3 expression was upregulated at 30 days, while both TNFAIP6 (60 days) and ST2 (60 days and 90 days) were downregulated with prebiotic/probiotic supplementation. Considering known biological actions, prebiotic/probiotic supplementation may have contributed to a transient reduction in adipose tissue inflammation and thus improved metabolic health and reduced chronic disease risk.

We found two additional mRNA (CMKLR1 and CD9) that were also differentially expressed with prebiotic/probiotic supplementation and related to adipose tissue inflammation; however, the magnitude of change was very small compared to those described above. Chemokine-like receptor 1 (CMKLR1) is a receptor for chemerin, a potent adipokine that is elevated with obesity [13, 15]. Adipose tissue macrophages that are CD9+ contribute to adipose tissue inflammation in obese individuals [14]. Based on their biological actions, one would assume that an increase in either CMKLR1 or CD9 would not be a beneficial effect. In the present study, both factors increased to a small degree at 30 days but then rapidly declined or remained stable at 60 days and 90 days. Given the short-term response and small differential expression, it is reasonable to speculate that these effects were likely not associated with increased risk and may be a result of the body adapting to the prebiotic/probiotic supplement.

We found significant differential expression of three mRNA (LTF, SOCS1, and SERPING1) that are linked to systemic inflammation. Metabolically unhealthy obese individuals have elevated LTF [5] and elevated SOCS1 [27]. Controlled weight loss is associated with reduced LTF [6]. Furthermore, elevated SOCS1 in obese individuals increased adipose tissue macrophage response to endotoxin, downstream pro-inflammatory cytokine production [21], and insulin resistance (1). The observed reduction in both LTF (90 days) and SOCS1 (60 days and 90 days) with prebiotic/probiotic supplementation would be considered a beneficial adaptation. SERPING1 encodes for C1 inhibitor and is negatively associated with incidence of LPS endotoxin shock in murine models [20]. Thus, SERPING1 differs from LTF and SOCS1 in that an increased expression would be considered a beneficial effect of supplement, which is what we found at 30 days in the present study. Collectively, the reduced LTF, reduced SOCS1, and increased SERPING1 may translate to a reduction in leukocyte response to LPS, reduced pro-inflammatory cytokine production, and reduced systemic inflammation.

Metabolic health is generally considered to be a precursory state that proceeds the onset and progression of chronic disease [19]. In the present study, prebiotic/probiotic supplementation was associated with change in 6 mRNA associated with reduced adipose tissue inflammation and reduced systemic inflammation. Based on the literature, we speculate that these changes support an improved metabolic health following 90 days of prebiotic/probiotic supplementation. Extending these findings further, we found significant change in 7 mRNA (CCL4, IL-18, ARG1, ATM, CD80, CEACAM6, and LAMP3) whose differential expression has been associated with various inflammation-associated chronic disease states. Elevated CCL4 [24] and IL-18 [26] result in elevated systemic inflammation and the onset and progression of type 2 diabetes mellitus. In the present study, we observed decreased differential expression of CCL4 and IL-18 at 60 days with prebiotic/probiotic supplementation, which given their associations should be associated with a reduced risk of type 2 diabetes mellitus.

Arginase 1 (ARG1), ATM, and CD80 are elevated with various forms of cardiovascular disease including the following: vascular dysfunction [28], coronary artery disease [22], and atherosclerosis [25] in obese individuals. In the present study, we found that prebiotic/probiotic supplementation reduced ARG1 (30 days), ATM (90 days), and CD80 (60 days), which may translate to a reduced cardiovascular disease risk. It was also interesting to note that the time course of change for these three indices was progressive where ARG1 was reduced first, followed by CD80 reduction, and finally with ATM reduction. It is possible that prebiotic/probiotic supplementation resulted in a coordinated response of these three mRNA. Knowing the nature of these responses, it is important for future studies to carefully select their mRNA biomarkers based on the length of the study. Specifically, ARG1 may be better for short-term studies (< 30 days), while ATM may be better for long-term studies (> 90 days).

Type 2 diabetes mellitus and cardiovascular disease are not the only chronic disease states associated with elevated systemic. LAMP3 is elevated in obese mice and associated with the progression of non-alcoholic fatty liver disease (NAFLD) [29], while CEASAM6 is elevated in Crohn disease patients and may be an index of colon inflammation [23]. In the present study, we observed significant reductions in LAMP3 (60 days) and CECAM6 (90 days). Both LAMP3 and CECAM6 were upregulated at 30 days, followed by suppression at 60 days and 90 days. This pattern of change was similar to that observed above (CMKLR1 and CD9) and may represent the biological adjustment period to starting a prebiotic/probiotic supplement. Unfortunately, we did not have a sampling interval between 30 days and 60 days, so we are unable to narrow down the length of initial adaptation in this trial. Determining the adjustment period may be the focus of future experimental design in this area.

In addition to the above significantly changed mRNA expression, we also found that the prebiotic/probiotic supplement differentially changed the expression of 6 mRNA (CSF1, SRC, ICAM4, CD24, CD274, CLEC6A) that based on the literature cannot be directly linked to either metabolic health, inflammation, and/or chronic disease. These mRNA are summarized in Fig. 4. As with any study, the present study is not without limitations. The small sample size could be considered a limitation; however, since we found significant differential expression of 21 mRNA with prebiotic/probiotic supplementation, we have at least a partial target response. In future studies with increased sample size, we will be able to narrow our RNA biomarker panel to focus on genes associated with or related to the ones that were changed in the present study. In future studies, we will also seek to link changes in mRNA and protein biomarkers. Being able to narrow down the biomarker targets represents a substantial time and cost-savings for our laboratory and others. It is important to acknowledge that it is possible we failed to identify all mRNA that were differentially expressed with prebiotic/probiotic supplementation because we may have committed a type 2 error, which will be addressed and confirmed in future studies. While the present study included a longer supplementation period than is normal in the literature, it was by no means long duration, so more research is needed to determine the stability of the observed responses in supplementation periods exceeding 90 days. The present study was not a controlled dietary intervention, and thus changes in participant dietary habits may have confounded the present finding. It is unknown if the observed changes are stable after prebiotic/probiotic supplementation is discontinued, so future studies may also seek to establish how discontinuation of supplementation influences metabolic health, inflammation, and chronic disease risk.

## Conclusion

The present study was completed to determine if 90 days of supplementation with a combination of prebiotic/probiotic could alter mRNA responsible for inflammation and chronic disease risk in weight stable overweight adults. Our key findings were that we identified 15 mRNA whose biological action was related to adipose tissue inflammation, systemic inflammation, and/or chronic disease and were improved with 90 days of prebiotic/probiotic supplementation. We identified an additional 6 mRNA that responded to prebiotic/probiotic supplementation but have not been previously linked to inflammation/chronic disease and may be considered novel outcome measures. Meta-analysis was used to confirm that the changes over time were associated with the prebiotic/probiotic supplement. The diurnal pattern of mRNA varied considerably, highlighting the importance of longer-term interventions to maximize the effectiveness of a prebiotic/probiotic supplement on various aspects of biological function. We believe the observed changes in VAT along with the 15 mRNA support the notion that inflammation and chronic disease risk can be reduced even in the absence of weight loss in free-living overweight adults. It is our hope that the key findings of the present study will provide critical information to support future interventions aimed at reducing chronic disease risk via a prebiotic/probiotic supplement.

## Methods

### Study participants

All the procedures described in the present study were reviewed and approved by the University of North Texas Institutional Review Board for Human Subject’s Research. Participants gave verbal and written informed consent, and procedures were conducted in accordance with the latest edition of the *Declaration of Helsinki* [30]. Exclusion criteria included body mass index less than 25 kg/m^2^, the presence of cardiovascular or metabolic disease (i.e., diabetes mellitus), current or previous (past 12 months) tobacco or e-cig user, regular user of dietary supplements that may reduce inflammation (i.e. curcumin, pomegranate polyphenols, MSM), individual consuming an atypical diet (i.e., ketogenic, paleo, vegan), use of antibiotics, probiotics, and/or anti-inflammatory medications in the previous 6 months. Participants were recruited from the DFW metroplex, which is a diverse community with good representation of the national population.

### Participant consort tracking

**Fig. 7:**
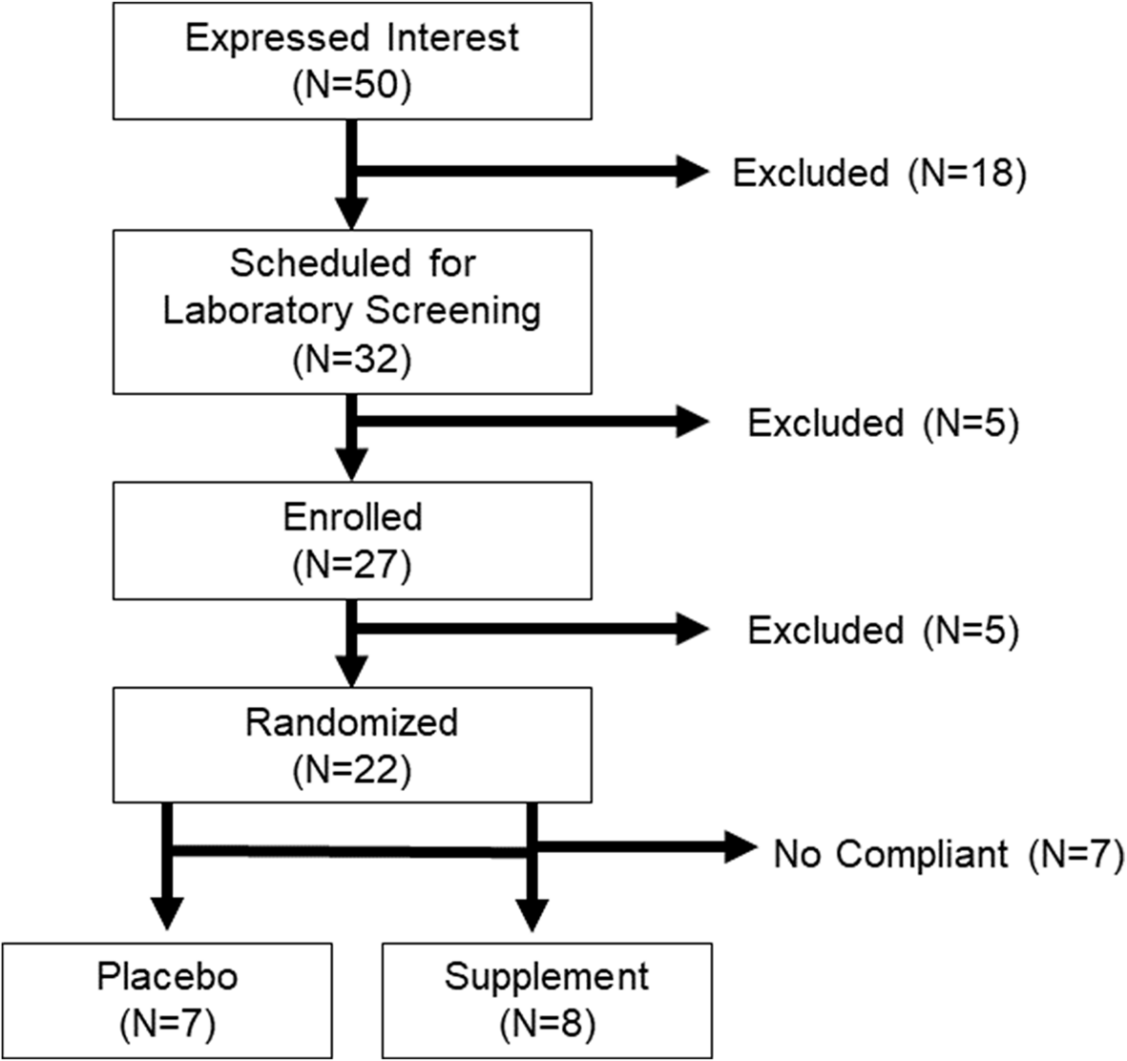
Consort diagram depicting progression from subject recruitment through study completion. Twenty-two participants were enrolled in the initial trial and fifteen completed all study requirements

Thirty-two people provided consent for this study. Twenty-seven people met all inclusion/exclusion criteria (5 could not be scheduled further due to time constraints unrelated to the study). Twenty-two participants were randomized in an equal split (*n* = 11) to each condition, and 15 participants completed all study requirements. Seven participants were excluded for missing study appointments. Participants who were dropped from the study for missing study appointments indicated that they had a personal scheduling conflict that did not allow them to continue their participation. No participants reported side effects associated with the supplement or study participation as a reason for not continuing. A detailed breakdown of participant consort tracking can be found in Fig. 7.

### Experimental design

An a priori sample size analysis was not conducted because we could not identify previously published data that would have provided data necessary for such an analysis. Despite this, we were able to estimate an approximate sample size using data from previously published studies in our laboratory [8, 31, 32]. Thus, an objective of the present study was to establish a data set that could support future study designs in this area. We used the gold standard double-blind, placebo-controlled design to maximize applicability to future studies.

Seven of the participants who completed all study requirements were in the placebo group, and 8 were in the supplement group. The supplement consisted of a prebiotic (PreticX® Xylooligosaccharide, 0.8 g/day, AIDP, City of Industry, CA, USA) and a probiotic (MegaDuo® *Bacillus subtilis* HU58 and *Bacillus coagulans* SC-208; 3 × 10^9^ CFU/day; Microbiome Labs, LLC; Phoenix, AZ, USA). The placebo was an equal weight of rice flour. Daily supplement (or placebo) dose was provided in three capsules, and supplementation began on day 1. Participants reported to the laboratory on four occasions: baseline (day 0), 30 days, 60 days, and 90 days. Participants were asked to bring their blister pack from the previous 30-day period to calculate compliance with the dosing requirements. When participants missed a dose during the study, they were asked to contact a member of the study staff. This information combined with the visual inspection of returned blister packs revealed that there was > 85% compliance with the supplement protocol with only two participants reporting missed doses. During the study, participants were advised not to change their diet or exercise habits to ensure a “free-living” intervention design.

At each appointment, participants provided a fasted blood sample and had their body composition and anthropometrics measured. At baseline, 30 days, and 60 days, they were provided with a 30-day supply of supplement in a blister pack. Appointments were scheduled between 0500 h and 1100 h, and participants were asked to verify that they had abstained from exercise for at least 12 h, and that they had not ingested anything other than water for at least 8 h prior to arriving at the laboratory.

### Body composition and anthropometrics

Height and weight were measured using a stadiometer and digital scale respectively (SECA, USA). Participants were asked to place their back and heels against the wall, and then the top of the stadiometer was placed on their head, and height was recorded. Participants were then asked to remove shoes/socks and items from their pockets to have their weight measured. Whole-body composition was measured using a DXA scan (GE Lunar iDXA, Chicago, IL, USA). Visceral adipose tissue (VAT) was calculated (Lunar enCore CoreScan v17). The DXA machine was calibrated daily according to the manufacture recommendations. The DXA software also estimates the resting metabolic rate (RMR) using the standard Harris-Benedict equation. RMR was only used as a descriptive variable to demonstrate the two groups were not different at baseline. All body composition values were expressed as a percentage change over time compared to baseline.

### Blood collection, RNA isolation, and mRNA expression analysis

Samples analysis was conducted in a manner consistent with previously published studies from our laboratory [31, 32]. Whole blood was collected from an antecubital vein in the arm into a 10 mL (2.5 mL blood + 6.5 mL PAXgene stabilizing solution) PAXgene RNA stabilizing vacutainer (PreAnalytiX; Hombrechtikon, Switzerland). Collected samples were mixed by inversion, stored at −20 °C for 24 h, followed by long-term storage at −80 °C until isolation and analysis. RNA was isolated as described previously [31, 32]. Briefly, total RNA was extracted using an automated (QIAcube; Qiagen, Hilden, Germany), commercially system (PAXgene Blood miRNA kit; PreAnalytiX). Isolated RNA was concentrated to 35 ng/μL using a commercially available spin column kit (RNA Clean and Concentrator Kit, Zymo Research, Irvine, CA, USA) prior to analysis. Total RNA was analyzed using a 574-plex Human Immunology Panel v 2.0, and raw image counts (RCC files) were obtained using a Sprint Profiler (nCounter; Nanostring, Seattle, WA, USA). In addition to the targets, specific internal positive/negative controls and housekeeping mRNA (EEF1G, RPL19, PPIA, HPRT1, TBP, TUBB, POLR1B, ABCF1, SDHA, GUSB, ALAS1, POLR2A, GAPDH, and OAZ1) were included with every RNA sample.

### Statistical methods

Data were analyzed by ROSALIND® (https://rosalind.onramp.bio/) with a HyperScale architecture developed by ROSALIND, Inc. (San Diego, CA, USA). Read distribution percentages, violin plots, identity heat maps, and sample multidimensional plots were generated as part of the quality control step (Fig. 7). Normalization, fold changes, and *p*-values were calculated using criteria provided by Nanostring. ROSALIND® follows the nCounter® Advanced Analysis protocol of dividing counts within a lane by the geometric mean of the normalizer probes from the same lane. Housekeeping probes that were used for normalization were selected based on the geNorm algorithm as implemented in the NormqPCR R library [33]. Fold changes and significance score (*p*-value) were calculated using the fast method as described in the nCounter® Advanced Analysis 2.0 User Manual (Nanostring). Gene clustering (based on direction and type of all signals on a pathway, the position, role, and type of every gene) of differentially expressed genes was determined using the partitioning around medoids method and the fpc R library [34]. Differential expression analysis was conducted comparing supplement to placebo at three time points (30, 60, and 90 days). The clusters of mRNA generated from the three differential analyses were used to conduct a ROSALIND® meta-analysis to identify specific mRNA that were changed over the course of the 90-day intervention with supplement compared to placebo. Supplement mRNA were expressed as log2 fold change from the corresponding placebo time point to normalize response to a 0 center and indicate direction of expression (i.e., up- vs. downregulated). Significant *p*-values (*p* < 0.05) were adjusted for multiple genetic comparisons using the Benjamini-Hochberg method of estimating false discovery rates [35]. Data demonstrating the relative distribution of gene expression via violin plots, heat map, and relative positive/negative control expression is presented in Fig. 6.

## Data Availability

The datasets used and/or analyzed during the current study are available from the corresponding author on reasonable noncommercial request.

## Abbreviations

ARG1: Arginase 1
ATM: ATM serine/threonine kinase
CD80: CD80 molecule
VAT: Visceral adipose tissue
GATA3: GATA binding protein 3
TNFAIP6: Tumor necrosis factor alpha-induced protein 6
ST2: Suppression of tumorigenicity 2
CMKLR1: Chemokine-like receptor 1
CD9: CD9 molecule
LTF: Lactotransferrin
SOCS1: Suppressor of cytokine signaling 1
SERPING1: Serpin peptidase inhibitor member 1
CCL4: Chemokine (C-C motif) ligand 4
IL-18: Interleukin-18
CEACAM6: Carcinoembryonic antigen-related cell adhesion molecule 6
LAMP3: Lysosomal-associated membrane protein 3
CSF1: Colony-stimulating factor 1
SRC: SRC proto-oncogene non-receptor tyrosine kinase
ICAM4: Intercellular adhesion molecule 4
CD24: CD24 molecule
CD274: CD274 molecule
CLEC6A: C-type lectin domain family 6 member A
